# Efficient Wideband Numerical Simulations for Nanostructures Employing a Drude-Critical Points (DCP) Dispersive Model

**DOI:** 10.1038/s41598-017-02194-1

**Published:** 2017-05-18

**Authors:** Qiang Ren, Jogender Nagar, Lei Kang, Yusheng Bian, Ping Werner, Douglas H. Werner

**Affiliations:** Department of Electrical Engineering, The Pennsylvania State University, University Park, PA, 16802 USA

## Abstract

A highly efficient numerical approach for simulating the wideband optical response of nano-architectures comprised of Drude-Critical Points (DCP) media (e.g., gold and silver) is proposed and validated through comparing with commercial computational software. The kernel of this algorithm is the subdomain level discontinuous Galerkin time domain (DGTD) method, which can be viewed as a hybrid of the spectral-element time-domain method (SETD) and the finite-element time-domain (FETD) method. An *hp*-refinement technique is applied to decrease the Degrees-of-Freedom (DoFs) and computational requirements. The collocated E-J scheme facilitates solving the auxiliary equations by converting the inversions of matrices to simpler vector manipulations. A new hybrid time stepping approach, which couples the Runge-Kutta and Newmark methods, is proposed to solve the temporal auxiliary differential equations (ADEs) with a high degree of efficiency. The advantages of this new approach, in terms of computational resource overhead and accuracy, are validated through comparison with well-known commercial software for three diverse cases, which cover both near-field and far-field properties with plane wave and lumped port sources. The presented work provides the missing link between DCP dispersive models and FETD and/or SETD based algorithms. It is a competitive candidate for numerically studying the wideband plasmonic properties of DCP media.

## Introduction

In recent years, the field of plasmonics has received considerable attention due to a wide variety of significant applications, including those that exploit surface plasmon polaritons (SPPs)^1–7^, optical metamaterials^8–12^ and nanoantennas^13–16^. In optics, metals, such as gold (Au) and silver (Ag), are wildly utilized for building nanoarchitectures due to their potential for plasmonic resonance. However, the substantial dispersion and loss of metals in the optical regime must be considered. This dispersion and loss can be incorporated into the mathematical expression of the permittivity, which is referred as the dispersive model. The classical and well-known models include Drude, Debye and Lorentz^17^, while some more efficient parameterizations of the dielectric property have been proposed such as the Drude-Lorentz and L4 models^18, 19^. Recent studies have shown that the Drude-Critical Points (DCP) model is more efficient and accurate when compared to the previous models over a wide frequency range^20, 21^, especially for the noble metals such as gold.

Due to the expeditious growth in nanotechnology, numerical simulation techniques for nano-architectures have recently begun to receive considerable attention. However, the time domain numerical techniques for DCP media, despite their potential advantages in wideband simulations, have not been addressed as extensively as those formulations based on the more classical models. There are mainly two reasons for this. First, the DCP model is relatively new and, therefore, comparatively less work has been done to implement it into time domain based numerical formulations. Second, unlike its frequency domain counterparts, in which the dispersive property can be simply incorporated into the permittivity, the time domain solver requires auxiliary differential equations or convolutions which are, computation-wise, much more complicated.

The state-of-the-art time domain numerical methods for general DCP geometries are mainly based on the FDTD method. Lu *et al*. utilized a 2D FDTD method to investigate the interaction between DCP nanostructures^22, 23^. Via *et al*. successfully implemented a recursive convolution (RC) 3D FDTD technique to model plasmonic structures and their interaction with light^24–26^. Chun *et al*.^27^ implemented the piecewise linear recursive convolution (PLRC) FDTD and auxiliary differential equation (ADE) FDTD method with two critical points to describe DCP media and carried out a detailed comparative study. A more general ADE FDTD, which allows an arbitrary number of Drude and critical point terms, was later introduced with perfectly matched layers (PML) to truncate the computational domain^28^. The DCP model was also incorporated into the frequency-dependent FDTD method using the trapezoidal recursive convolution technique^29^. A comprehensive study on the convergence and accuracy of FDTD for nanoplasmonic structures has been reported in ref. 30. The development of these approaches has significantly improved simulation techniques for plasmonic media; however, they also inherit the deficiencies of conventional FDTD, including the staircase error for curved surfaces, difficulties in modeling thin structures and a relatively large spatial sampling density requirement when compared to methods allowing high order basis functions, such as the finite element method (FEM) and the spectral element method (SEM).

Unlike FDTD, the finite element time domain (FETD) method, which utilizes an unstructured mesh, is free of any staircase error. In addition, it also inherits the merits of time domain approaches, such as wideband simulation in one shot and the ease of solving nonlinear problems. However, the application of FETD for DCP media has not been studied as thoroughly as it has for the FDTD method. Prokopeva *et al*. developed an FETD time integration scheme for DCP media^31^ with 2D validation, where the spatial discretization along with the matrix assembling were performed using a commercial finite element code (COMSOL multiphysics).

**Figure 1:**
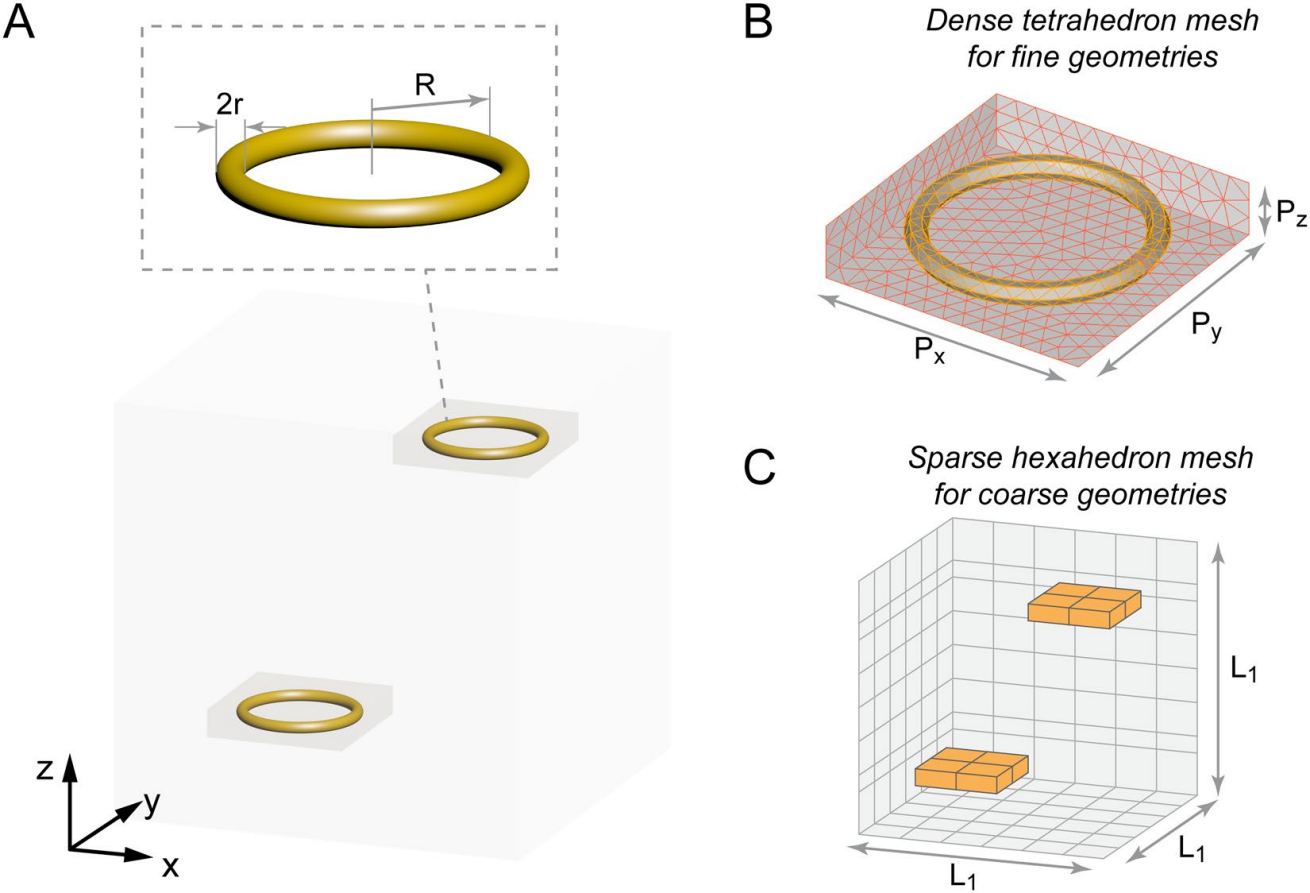
Geometry and mesh of the nanoloop coupling example. (**A**) The centers of the two gold nanoloops are located at [−500, −500, −900] nm and [500, 500, 900] nm, respectively. The radius *R* is 400 nm and the thickness, 2*r*, is 80 nm. (**B**) The nanoloops and their surrounding spaces are separated from the entire computational region and meshed with low-order tetrahedrons to capture the detailed geometry information. (Only the mesh on the surfaces are shown while the volumetric mesh is hidden to improve visual clarity. Three of the exterior surfaces are set to be transparent to reveal the nanoloop). P_*x*_ = 1000 nm; P_*y*_ = 1000 nm and P_*z*_ = 200 nm. (**C**) The remaining of the physical region is meshed with high-order hexahedrons. The space inside the yellow boxes are vacuum as they have already been meshed with tetrahedrons in (**B**). L_1_ = 3000 nm. The shared interfaces (yellow surfaces) uses rectangles mesh on one side while the other side uses triangles. Ren *et al*. have introduced the technique, which is also used in this work, to solve the energy communication across these non-conformal interfaces^32^.

In this paper, we propose a 3D DGTD (FETD based) approach for simulating the wideband response of DCP dispersive nanostructures. The advantages of this new algorithm are threefold. *First*, it employs domain decomposition and *hp*-refinement, a framework that is similar to the previous **EB** scheme subdomain level DGTD method^32–35^. The mixed **EB** scheme, which is free of the spurious modes, is an efficient realization of the discrete Hodge operator^36–40^. Domain decomposition can divide one computationally intensive problem into a few smaller ones with lower computational complexity, while the Riemann solver is utilized to link them. The *hp*-refinement uses dense meshed low-order tetrahedron elements (*h*-refinement) to discretize the geometrically fine structures and adapts high-order hexahedron elements (*p*-refinement) to mesh the spacious homogeneous background and the coarse structures. In this fashion, the geometrical accuracy and the efficiency in the Degrees of Freedom (DoF) are maintained simultaneously. To elucidate this procedure, a schematic of the meshing strategy of two coupled nanoloops, a sophisticated example discussed in detail later, is illustrated in Fig. 1. In particular, as shown in Fig. 1(B), due to the curved surfaces and relatively thin structure (2*r*), the nanoloops and their surrounding space are separated and meshed with dense low-order tetrahedrons to capture the fine geometries. For the remaining free space, a high-order hexahedron mesh is preferred due to the spatial sampling efficiency, as shown in Fig. 1(C). A Riemann solver is applied to fulfill the energy communication between the hexahedron subdomain and the tetrahedron subdomains. It is worth pointing out that all the mesh plots in this paper only show the mesh on the surfaces of the objects and the volumetric meshes are hidden to improve visual clarity. *Second*, the auxiliary differential equations (ADEs) employed to represent the constitutive relations of the dispersive media adopt E-J collocation to transform the inversion of matrices into efficient vector manipulation. *Third*, a hybrid scheme is proposed for the time integration. The second order Runge-Kutta (mid-point) method^33, 41^ is used to update the **E** and **B** fields as well as the polarization currents of the Drude model. The Newmark method^42^ is chosen to update the polarization currents of the Lorentzian resonances corresponding to the critical points of the band transitions. The two methods are arranged in a proper updating sequence in the coupled system to achieve accurate results with efficacy.

The proposed method is applied to three examples of DCP gold nano-architectures: the near-field response of a cube, the far-field radar cross section (RCS) of a split ring resonator (SRR), and the mutual coupling between two nanoloops. Besides good agreement, a comparison to the results obtained from widely used commercial software packages including ANSYS Electronics Desktop (AEDT) and FEKO, indicates that the proposed method is more computationally efficient as well as requires less CPU time and memory.

## Theory and Methods

### System Equations (Maxwell’s Equations and Auxiliary Differential Equations)

The DCP model under consideration in this study was first derived by Etchegoin *et al*.^20^ and extended by McKinley *et al*.^13^, in which the relative permittivity is represented as:

1 $${\varepsilon }_{r}=1-\frac{{f}_{0}{\omega }_{p}^{2}}{\omega }(\frac{1}{\omega -j2{{\rm{\Gamma }}}_{0}}+\frac{\alpha }{\omega -j\beta 2{{\rm{\Gamma }}}_{0}})+\sum _{m=1}^{M}\frac{{f}_{m}{\omega }_{p}^{2}}{2{\omega }_{m}}(\frac{{e}^{j\frac{\pi }{{\gamma }_{m}}}}{{\omega }_{m}-\omega +j{{\rm{\Gamma }}}_{m}}+\frac{{e}^{-j\frac{\pi }{{\gamma }_{m}}}}{{\omega }_{m}+\omega -j{{\rm{\Gamma }}}_{m}})$$

The associated susceptibility is defined by:

2 $$\chi (\omega )=\frac{{f}_{0}{\omega }_{p}^{2}}{j\omega (2{{\rm{\Gamma }}}_{0}+j\omega )}+\frac{\alpha {f}_{0}{\omega }_{p}^{2}}{j\omega (2\beta {{\rm{\Gamma }}}_{0}+j\omega )}+\sum _{m=1}^{M}\frac{{f}_{m}{\omega }_{p}^{2}[\cos (\frac{\pi }{{\gamma }_{m}})+\frac{({{\rm{\Gamma }}}_{m}+j\omega )}{{\omega }_{m}}\,\sin (\frac{\pi }{{\gamma }_{m}})]}{({\omega }_{m}^{2}+{{\rm{\Gamma }}}_{m}^{2})+2j{{\rm{\Gamma }}}_{m}\omega -{\omega }^{2}}$$

The first two terms correspond to the standard two-pole Drude model. The damping coefficients are 2Γ_0_ and 2*β*Γ_0_, while the plasma frequencies are $\sqrt{{f}_{0}}{\omega }_{p}$ and $\sqrt{\alpha {f}_{0}}{\omega }_{p}$. The third term is a summation of Lorentzian resonances with critical transitions, where ${\omega }_{m}$ are the critical points, *f*_*m*_ are the quantum probabilities of transition and Γ_*m*_ are the Lorentz broadening terms.

We can define the ADEs for the polarization currents associated with the two Drude terms and the Lorentzian terms as:

3 $${{\bf{J}}}_{D,1}=\frac{{f}_{0}{\omega }_{p}^{2}}{2{{\rm{\Gamma }}}_{0}+j\omega }{\bf{E}};\,{{\bf{J}}}_{D,2}=\frac{\alpha {f}_{0}{\omega }_{p}^{2}}{2\beta {{\rm{\Gamma }}}_{0}+j\omega }{\bf{E}};\,{{\bf{J}}}_{L,m}=\frac{{\alpha }_{m}(j\omega )+{\beta }_{m}{(j\omega )}^{2}}{({\omega }_{m}^{2}+{{\rm{\Gamma }}}_{m}^{2})+2j{{\rm{\Gamma }}}_{m}\omega -{\omega }^{2}}{\bf{E}}$$

where

4 $${\alpha }_{m}={f}_{m}{\omega }_{p}^{2}\,\cos (\frac{\pi }{{\gamma }_{m}})+\frac{{f}_{m}{\omega }_{p}^{2}{{\rm{\Gamma }}}_{m}}{{\omega }_{m}}\,\sin (\frac{\pi }{{\gamma }_{m}});\,{\beta }_{m}=\frac{{f}_{m}{\omega }_{p}^{2}}{{\omega }_{m}}\,\sin (\frac{\pi }{{\gamma }_{m}})$$

Thus Maxwell’s equations for the DCP medium can be expressed in terms of the field variables **E** and **B** and the polarization currents given in Eq. 3:

5 $${\varepsilon }_{0}\frac{\partial {\bf{E}}}{\partial t}=\frac{1}{{\mu }_{0}}\nabla \times {\bf{B}}-{{\bf{J}}}_{D,1}-{{\bf{J}}}_{D,2}-\sum _{m=1}^{M}{{\bf{J}}}_{L,m}$$

6 $$\frac{\partial {\bf{B}}}{\partial t}=-\,\nabla \times {\bf{E}}$$

To solve Eqs 5 and 6, divergence conforming basis functions, denoted by **Ψ**, are used to discretize **B**, while curl conforming basis functions, denoted by **Φ**, are used to discretize **E**, **J**_*D*,1_, **J**_*D*,2_ and **J**_*L*,*m*_. The expressions and field distributions of the 3D curl- and divergence-conforming basis functions can be found in refs 33 and 35.

Using **Φ** and **Ψ** to test Eqs 5 and 6, respectively, and applying the Riemann solver to treat the numerical flux between adjacent subdomains^33^, the discretized system equation for the i*th* subdomain can be obtained as:

7 $${{\bf{M}}}^{i}\frac{d{{\bf{u}}}^{i}}{dt}=-\,{{\bf{C}}}^{i}({{\bf{j}}}_{D,1}^{i}+{{\bf{j}}}_{D,2}^{i}+\sum _{m=1}^{M}{{\bf{j}}}_{L,m}^{i})+{{\bf{L}}}^{ii}{{\bf{u}}}^{i}+\sum _{j=1,\,j\ne i}^{{N}_{s}}{{\bf{L}}}^{ij}{{\bf{u}}}^{j}+{{\bf{j}}}^{i}$$

where ${{\bf{u}}}^{i}=[{{\bf{e}}}^{i};{{\bf{b}}}^{i}]$ and ${{\bf{u}}}^{j}=[{{\bf{e}}}^{j};{{\bf{b}}}^{j}]$ are the unknown field vectors for the i*th* and the j*th* (local and adjacent) subdomains, respectively. **j** ^*i*^ is the vector for the impressed current, **j**_*D*,1_, **j**_*D*,2_ and **J**_*L*,*m*_ are the unknown vectors for the polarization currents, and *N*_*s*_ is the number of subdomains.

We assume the sizes of **u** ^*i*^, **j**_*D*,1_, **j**_*D*,2_ and **J**_*L*,*m*_ are *N* × 1, and the set of DoF indices of the i*th* subdomain are $U=\{1,2,3,\ldots ,N\}$. The indices of the unknowns for the polarization currents form a subset of *U*, denoted by *U*′. If we assume the size of *U*′ is *N*′, then the size of the matrix **C** ^*i*^ is *N* × *N*′, where the elemental form is an inner product over the volume of an element *V*_*e*_:

8 $${{\bf{C}}}_{pq}^{i}={\langle {\varphi }_{p},{\varphi }_{q}\rangle }_{{V}_{e}}\,p,q\in U^{\prime} $$

Similarly, **Φ** is also used to test Eq. 3. With the help of E-J collocation^43, 44^ (the system matrices are identical and can be canceled) and conversion to the time domain, it follows from Eqs 9 and 10 that the auxiliary equations only require vector manipulations:

9 $$\frac{d{{\bf{j}}}_{D,1}^{i}}{dt}+2{{\rm{\Gamma }}}_{0}{{\bf{j}}}_{D,1}^{i}={f}_{0}{\omega }_{p}^{2}{\tilde{{\bf{e}}}}^{i};\,\frac{d{{\bf{j}}}_{D,2}^{i}}{dt}+2\beta {{\rm{\Gamma }}}_{0}{{\bf{j}}}_{D,2}^{i}=\alpha {f}_{0}{\omega }_{p}^{2}{\tilde{{\bf{e}}}}^{i}$$

10 $$\frac{{d}^{2}{{\bf{j}}}_{L,m}^{i}}{d{t}^{2}}+2{{\rm{\Gamma }}}_{m}\frac{d{{\bf{j}}}_{L,m}^{i}}{dt}+({\omega }_{p}^{2}+{{\rm{\Gamma }}}_{m}^{2}){{\bf{j}}}_{L,m}^{i}={\alpha }_{m}\frac{d{\tilde{{\bf{e}}}}^{i}}{dt}+{\beta }_{m}\frac{{d}^{2}{\tilde{{\bf{e}}}}^{i}}{d{t}^{2}}$$

where ${\tilde{{\bf{e}}}}^{i}\subset {{\bf{e}}}^{i}$ is a *N*′ × 1 vector corresponding to the electric field unknowns in the DCP medium.

### Time Integration Scheme

Equtions 7 and 9 are first order PDEs, and they can be solved by the explicit second order Runge-Kutta (mid-point) method. The tableau of coefficients is:

The Newmark-beta method (*β* = 0) is employed to solve Eq. 10, which is a second order PDE. Assuming the time step size is Δ*t* and evaluating Eq. 10 at $t=(n-\frac{1}{2}){\rm{\Delta }}t$, then ${{\bf{j}}}_{L,m}^{i}$ at *t* = nΔ*t* (denoted as ${{{\bf{j}}}_{L,m}^{i}|}_{n}$ for brevity) can be updated as shown below:

11 $${{{\bf{j}}}_{L,m}^{i}|}_{n}={{\bf{M}}}_{m}^{-1}[{{\bf{A}}}_{m}{{{\bf{j}}}_{L,m}^{i}|}_{(n-\frac{1}{2})}+{{\bf{B}}}_{m}{{{\bf{j}}}_{L,m}^{i}|}_{(n-1)}+{{\bf{X}}}_{m}\,{\frac{d{\tilde{{\bf{e}}}}^{i}}{dt}|}_{(n-\frac{1}{2})}+{{\bf{Y}}}_{m}\,\frac{{\frac{d{\tilde{{\bf{e}}}}^{i}}{dt}|}_{n}-{\frac{d{\tilde{{\bf{e}}}}^{i}}{dt}|}_{(n-1)}}{{\rm{\Delta }}t}]$$

where ${{\bf{M}}}_{m}=[\frac{4}{{({\rm{\Delta }}t)}^{2}}+\frac{2{\Gamma }_{m}}{{\rm{\Delta }}t}]{{\bf{I}}}_{N^{\prime} }$, ${{\bf{A}}}_{m}=[\frac{4}{{({\rm{\Delta }}t)}^{2}}-({\omega }_{p}^{2}+{{\rm{\Gamma }}}_{m}^{2})]{{\bf{I}}}_{N^{\prime} }$, ${{\bf{B}}}_{m}=[-\frac{4}{{({\rm{\Delta }}t)}^{2}}+\frac{2{\Gamma }_{m}}{{\rm{\Delta }}t}]{{\bf{I}}}_{N^{\prime} }$, ${{\bf{X}}}_{m}={\alpha }_{m}{{\bf{I}}}_{N^{\prime} }$, ${{\bf{Y}}}_{m}={\beta }_{m}{{\bf{I}}}_{N^{\prime} }$ and **I**_N′_ is an identity matrix of size *N*′ × *N*′.

Assuming ${{\bf{u}}|}_{n}$, ${{{\bf{j}}}_{L,m}|}_{(n-\frac{1}{2})}$, ${{{\bf{j}}}_{D,1}|}_{n}$ and ${{{\bf{j}}}_{D,2}|}_{n}$ for all subdomains have already been obtained and ${\frac{d{{\bf{u}}}^{i}}{dt}|}_{(n-\frac{1}{2})}$ and ${\frac{d{{\bf{u}}}^{i}}{dt}|}_{(n-1)}$ are also stored, Eq. 11 can be substituted into Eq. 7 to evaluate ${\frac{d{{\bf{u}}}^{i}}{dt}|}_{n}$:

12 $$\begin{array}{rcl}[{{\bf{M}}}^{i}+\frac{{\sum }_{m=1}^{M}{{\bf{C}}}^{i}{{\bf{M}}}_{m}^{-1}{{\bf{Y}}}_{m}{{\bf{P}}}^{i}}{{\rm{\Delta }}t}]{\frac{d{{\bf{u}}}_{i}}{dt}|}_{n} & = & {{\bf{L}}}^{ii}{{{\bf{u}}}^{i}|}_{n}+\sum _{j=1,\,j\ne i}^{{N}_{s}}{{\bf{L}}}^{ij}{{{\bf{u}}}^{j}|}_{n}+{{{\bf{j}}}^{i}|}_{n}-{{\bf{C}}}^{i}({{{\bf{j}}}_{D,1}|}_{n}+{{{\bf{j}}}_{D,2}|}_{n})\\  &  & -\sum _{m=1}^{M}({{\bf{C}}}^{i}{{\bf{M}}}_{m}^{-1}{{\bf{A}}}_{m}{{{\bf{j}}}_{L,m}^{i}|}_{(n-\frac{1}{2})}+{{\bf{B}}}_{m}{{{\bf{j}}}_{L,m}^{i}|}_{(n-1)}\\  &  & +{{\bf{X}}}_{m}\,{\frac{d{\tilde{{\bf{e}}}}^{i}}{dt}|}_{(n-\frac{1}{2})}-\frac{{{\bf{Y}}}_{m}{{\bf{P}}}^{i}}{{\rm{\Delta }}t}{\frac{d{\tilde{{\bf{e}}}}^{i}}{dt}|}_{(n-1)})\end{array}$$

where **P** ^*i*^ is a *N*′ × *N* matrix which isolates the **E** field unknowns in the DCP medium volume. The matrix $[{{\bf{M}}}^{i}+\frac{{\sum }_{m=1}^{M}{{\bf{C}}}^{i}{{\bf{M}}}_{m}^{-1}{{\bf{Y}}}_{m}{{\bf{P}}}^{i}}{{\rm{\Delta }}t}]$ can be pre-factorized (i.e. by using an LU decomposition) and used for the subsequent back-substitution in every time step. Then ${{{\bf{j}}}_{L,m}^{i}|}_{n}$ can be updated via Eq. 11 after ${\frac{d{{\bf{u}}}^{i}}{dt}|}_{n}$ is obtained.

Since ${{{\bf{e}}}^{i}|}_{n}$, ${{{\bf{j}}}_{D,1}|}_{n}$ and ${{{\bf{j}}}_{D,2}|}_{n}$ are known, then ${\frac{d{{\bf{j}}}_{D,1}^{i}}{dt}|}_{n}$ and ${\frac{d{{\bf{j}}}_{D,2}^{i}}{dt}|}_{n}$ can be evaluated from Eq. 9 as follows:

13 $${\frac{d{{\bf{j}}}_{D,1}^{i}}{dt}|}_{n}=-\,2{{\rm{\Gamma }}}_{0}{{{\bf{j}}}_{D,1}^{i}|}_{n}+{f}_{0}{\omega }_{p}^{2}{{\tilde{{\bf{e}}}}^{i}|}_{n};\,{\frac{d{{\bf{j}}}_{D,2}^{i}}{dt}|}_{n}=-\,2\beta {{\rm{\Gamma }}}_{0}{{{\bf{j}}}_{D,2}^{i}|}_{n}+\alpha {f}_{0}{\omega }_{p}^{2}{{\tilde{{\bf{e}}}}^{i}|}_{n}$$

The intermediate values of **u** ^*i*^, ${{\bf{j}}}_{D,1}^{i}$ and ${{\bf{j}}}_{D,2}^{i}$ used in the Runge-Kutta time stepping can be obtained by:

14 $${{\bf{v}}}_{u}={{{\bf{u}}}^{i}|}_{n}+\frac{{\rm{\Delta }}t}{2}{\frac{d{{\bf{u}}}^{i}}{dt}|}_{n};\,{{\bf{v}}}_{D,k}^{i}={{{\bf{j}}}_{D,k}^{i}|}_{n}+\frac{{\rm{\Delta }}t}{2}{\frac{d{{\bf{j}}}_{D,k}^{i}}{dt}|}_{n}(k=1,2)$$

If we change *n* to $(n+\frac{1}{2})$ in Eqs 12 and 14, and replace ${{{\bf{j}}}_{D,1}|}_{n}$, ${{{\bf{j}}}_{D,2}|}_{n}$ and ${{{\bf{u}}}^{i}|}_{n}$ with ${{\bf{v}}}_{D,1}^{i}$, ${{\bf{v}}}_{D,2}^{i}$ and **v**_*u*_, respectively, ${\frac{d{{\bf{u}}}^{i}}{dt}|}_{(n+\frac{1}{2})}$, ${\frac{d{{\bf{j}}}_{D,1}^{i}}{dt}|}_{(n+\frac{1}{2})}$ and ${\frac{d{{\bf{j}}}_{D,2}^{i}}{dt}|}_{(n+\frac{1}{2})}$ can be calculated. Then all the field unknowns can be updated to a new time step as in Eqs 15 and 16:

15 $${{{\bf{j}}}_{L,m}^{i}|}_{(n+\frac{1}{2})}={{\bf{M}}}_{m}^{-1}\,[{{\bf{A}}}_{m}{{{\bf{j}}}_{L,m}^{i}|}_{n}+{{\bf{B}}}_{m}{{{\bf{j}}}_{L,m}^{i}|}_{(n-\frac{1}{2})}+{{\bf{X}}}_{m}\,{\frac{d{\tilde{{\bf{e}}}}^{i}}{dt}|}_{n}+{{\bf{Y}}}_{m}\,\frac{{\frac{d{\tilde{{\bf{e}}}}^{i}}{dt}|}_{(n+\frac{1}{2})}-{\frac{d{\tilde{{\bf{e}}}}^{i}}{dt}|}_{(n-\frac{1}{2})}}{{\rm{\Delta }}t}]$$

16 $${{{\bf{u}}}^{i}|}_{(n+1)}={{{\bf{u}}}^{i}|}_{n}+{\rm{\Delta }}t{\frac{d{{\bf{u}}}^{i}}{dt}|}_{(n+\frac{1}{2})};\,{{{\bf{j}}}_{D,k}^{i}|}_{(n+1)}={{{\bf{j}}}_{D,k}^{i}|}_{n}+{\rm{\Delta }}t{\frac{d{{\bf{j}}}_{D,k}^{i}}{dt}|}_{(n+\frac{1}{2})}(k=1,2)$$

To summarize, within each time step, the polarization currents from the Lorentzian critical points are updated twice using the Newmark-beta method, while the electric field, magnetic flux and polarization current from the Drude component are updated with a second order explicit Runge-Kutta method, thus the overall time discretization retains second order accuracy.

## Numerical Results and Discussions

### Validation Case with Near-field Response Comparison to Commercial Software

**Figure 2:**
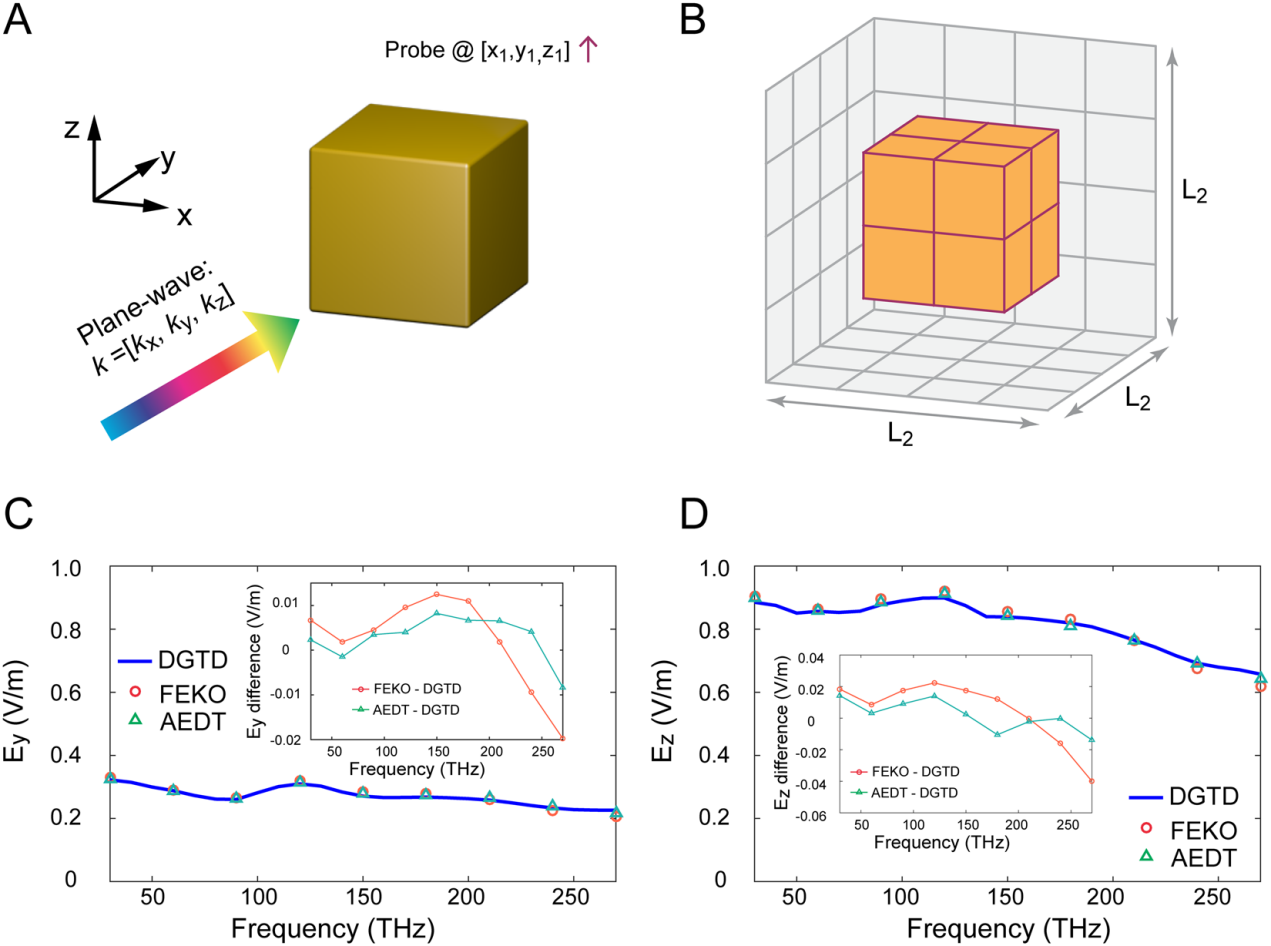
Geometry, mesh and results of validation case. (**A**) The center of the DCP gold cube is located at the origin and the associated edge length is 2000 nm. The excitation source is a wideband plane wave (approximately from 0 to 315 THz), the propagation and E field vectors are [0.6124, 0.6124, 0.5] and [0.35354, 0.35354, −0.86603], respectively. The probe is located at [1500, 1500, 1500] nm. (**B**) High order hexahedron mesh of physical region (the gold cube and its surrounding space), where L_2_ = 4000 nm. (**C**) Comparison of E_*y*_ at the probe, (**D**) Comparison of E_*z*_ at the probe.

To prove the effectiveness of the spatial discretization and time stepping schemes, the near-field response of a gold cube under an incident plane wave illumination (as illustrated in Fig. 2(A)) is considered as a validation case. The gold is described by a DCP model^13^ with the parameters listed in Table 1, where the conversion factor between eV and frequency is 4.13821 × 10^−15^.

**Table 1:** Parameters for gold based on DCP model in Eq. 1.

*f*_0_	*f*_1_	*f*_2_	*f*_3_	Γ_0_	Γ_1_	Γ_2_	Γ_3_	
0.37	0.20	0.35	0.6	0.005 eV	0.60 eV	1.1 eV	2.2 eV	
*α*	*β*	*γ*_1_	*γ*_2_	*γ*_3_	ω_1_	ω_2_	ω_3_	ω_*p*_
1.54	13.08	4.0	4.0	4.0	2.62 eV	3.7 eV	7.0 eV	9.0 eV

The center of this gold cube is located at the origin, and its edge length is 2 µm. The plane wave propagation vector is k = [k_*x*_, k_*y*_, k_*z*_] = [0.6124, 0.6124, 0.5] and the electric field polarization vector is [0.35354, 0.35354, −0.86603]. The source type utilized for the wideband excitation plane wave is the 1st order derivative of the Blackman-Harris window (BHW)^45^ pulse with a characteristic frequency of 100 THz (−30 dB frequency range is about [0, 315 THz]). For sampling the near-zone electric fields, a probe is located at $[{x}_{1},{y}_{1},{z}_{1}]=[1.5,1.5,1.5]$ µm. The physical region (*L*_2_ = 4 µm) consists of the gold cube and its surrounding free space as shown in Fig. 2(B). Each hexahedron represents a 4th-order element, and the edge length is 1 µm. To truncate the physical region, one layer of 4th-order hexahedrons are extruded to form a perfectly matched layer (PML) (not shown) to absorb the outgoing wave. The PML formulations employed in this paper can be found in ref. 33, and we will not derive them in detail here.

The total number of simulation steps is 2000 with an interval of 0.05 fs. The fields recorded at the probe are normalized and then transformed from time domain to the frequency domain. The same case is simulated using the commercial software FEKO (frequency domain integral equation solver based) and AEDT (frequency domain finite element solver based) from 30 THz to 270 THz with an interval of 30 THz (9 frequency points in total). The y and z components of the electric field at the probe are compared with results from AEDT and FEKO, as illustrated in Fig. 2(C) and (D). The maximal differences between the commercial software and DGTD are observed to be only about 0.02 V/m and 0.04 V/m (relative errors of 10% and 6.8%), respectively. This good agreement validates the spatial discretization and time integration schemes for DCP media. It is worth noting that the numerical error obviously increases at the higher frequency range (200 THz–250 THz). The reason for this is twofold: first, in the DGTD method, the low spatial sampling density at high frequency will result in relatively less accurate results compared to those for low frequencies; second, the ultra-wideband excitation source has relatively smaller energy in the high frequency range, consequently, the accuracy is more sensitive to the numerical errors.

### Radar Cross Section (RCS) Calculation of Split Ring Resonator (SRR) with DCP Gold

**Figure 3:**
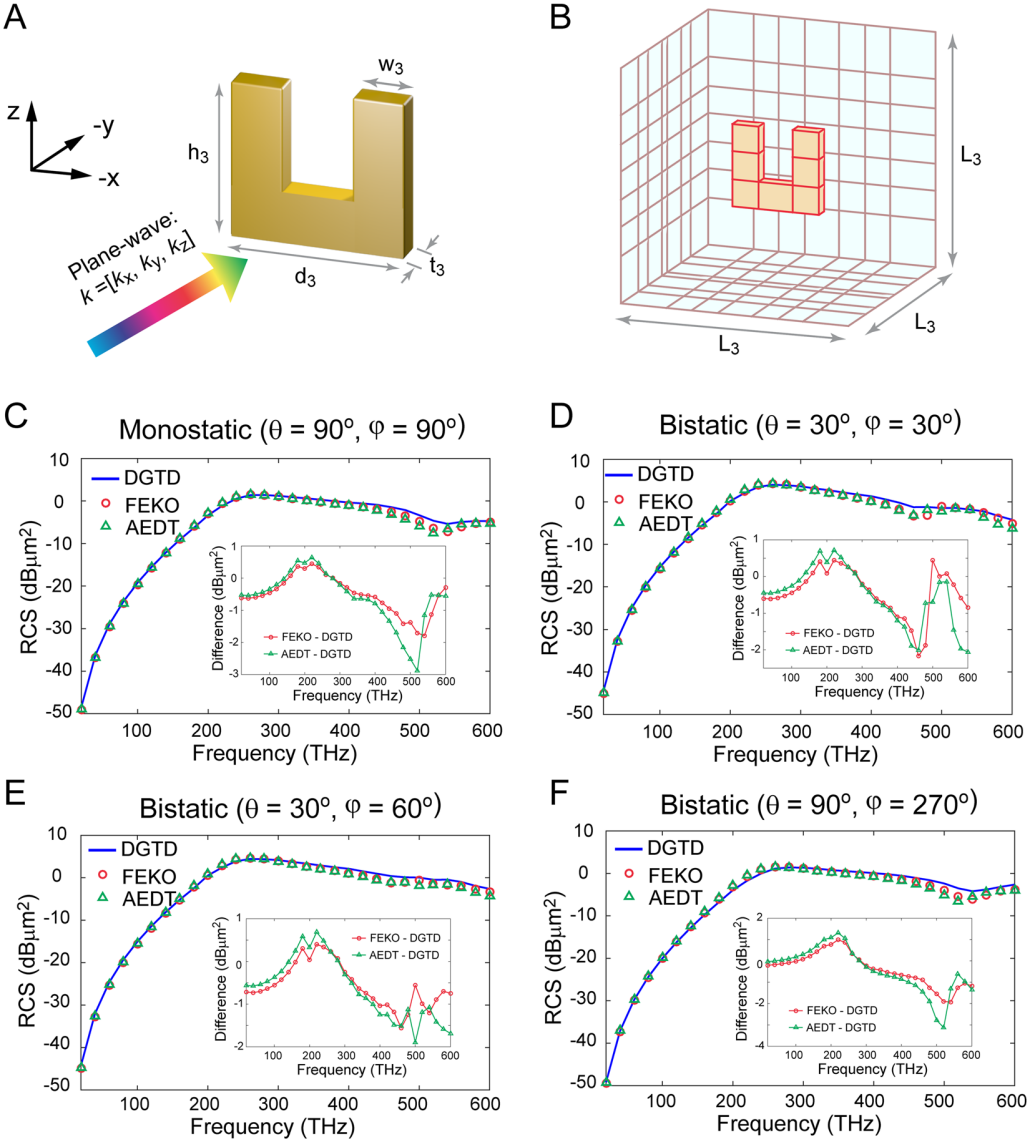
Geometry, mesh and results of SRR case. (**A**) The center of the DCP gold SRR is located at the origin. *d*_3_ = 380 nm, *h*_3_ = 350 nm, *w*_3_ = 115 nm and *t*_3_ = 60 nm. The excitation source is a wideband plane wave (approximately from 0 to 630 THz), the propagation and E field vectors are [0, −1, 0] and [0, 0, 1], respectively. (**B**) High order hexahedron mesh of physical region (the gold SRR and its surrounding space) with *L*_3_ = 2000 nm. (**C**) Comparison of monostatic RCS (θ = 90°, ϕ = 90°). (**D**) Comparison of bistatic RCS (θ = 30°, ϕ = 30°). (**E**) Comparison of bistatic RCS (θ = 30°, ϕ = 60°). (**F**) Comparison of bistatic RCS (θ = 90°, ϕ = 270°).

The SRR is a typical metamaterial structure that is widely used for different purposes such as achieving a magnetic response at optical frequencies. To test the efficiency of the proposed method, the RCS of a SRR composed of DCP gold is simulated. Figure 3(A) shows the structure of the SRR^46^, where *d*_3_ = 380 nm, *h*_3_ = 350 nm, *w*_3_ = 115 nm and *t*_3_ = 60 nm. The edge length of the physical region (SRR and its surrounding space) is *L*_3_ = 2 µm and the corresponding mesh is illustrated in Fig. 3(B).

The plane wave propagation vector is k = [k_*x*_, k_*y*_, k_*z*_] = [0, −1, 0] and the electric field polarization vector is [0, 0, 1]. The source type utilized for the wideband excitation plane wave corresponds to the 1st order derivative of BHW pulse with a characteristic frequency of 200 THz. The total simulation window is 50 fs with 5000 uniform time steps, leading to a resolution of 20 THz in the frequency spectrum.

The same case is also simulated in AEDT and FEKO. The frequencies range from 20 THz to 600 THz with a step size of 20 THz. In the DGTD method, the electric and magnetic fields are recorded at the six surfaces of a box with the edge length of 1.2 µm. After employing a near-to-far-field transformation, the monostatic RCS (θ = 90°, ϕ = 90°) and the bistatic RCS for (θ = 30°, ϕ = 30°), (θ = 30°, ϕ = 60°) and (θ = 90°, ϕ = 270°) are calculated in the DGTD method over the frequencies from 20 THz to 600 THz, and then compared to AEDT and FEKO. Good agreement is achieved as illustrated in Fig. 3(C–F). For the bistatic RCS at (θ = 30°, ϕ = 30°) and (θ = 30°, ϕ = 60°), the maximal difference is only approximately 2 dB between commercial software and the DGTD method. For the monostatic RCS (θ = 90°, ϕ = 90°) and the bistatic RCS at (θ = 90°, ϕ = 270°), the maximal differences are about 2 dB and 3 dB for FEKO and AEDT, respectively.

The DGTD code, AEDT and FEKO were all run on the same desktop (Intel XEON E5-2609 2.5-GHz four-core processor with 24 GB memory). The proposed DGTD method outperforms AEDT and FEKO in both time and peak memory consumption as demonstrated in Table 2. The reason for the advantages are four-fold: first, DGTD is a time domain approach and it can process the wideband analysis with just one simulation; second, high-order hexahedron elements employed in the DGTD method reduce the DoF, the total unknowns is smaller (only 79k) than that of AEDT which uses a low-order tetrahedron mesh over the computational region; third, the system matrices of the DGTD method are sparse, thus they are easier to solve than the dense matrices from FEKO; and fourth, the proposed Runge-Kutta and Newmark hybrid time integration scheme is efficient, it only takes 0.097 s for each time step.

**Table 2:** Computational overhead comparison for the SRR case between the DGTD method, AEDT and FEKO.

	FEKO	AEDT	DGTD	Gain wrt FEKO/AEDT
Total Time (s)	2159.9	654	576.6	3.75/1.13
Peak Memory (MB)	581.9	1710	455	1.28/3.76
Time Steps	N/A	N/A	5000	N/A
Time Interval	N/A	N/A	0.01 fs	N/A
Cell Size (nm)	Triangle 20	Tetrahedron 40	Brick 120 × 120 × 60	N/A
DoFs	8646	368078	78752	0.109/4.673
Element Number	2882	58121	406	7.1/143.2

### Mutual Coupling of DCP Nanoloops

Next, the mutual coupling between two nanoloops with DCP gold is studied with the proposed method to further exhibit its advantages. The centers of the two nanoloops are located at [−500, −500, −900] nm and [500, 500, 900] nm, respectively. The radius *R* of each nanoloop is 400 nm and the thickness, 2*r*, is 80 nm as shown in Fig. 1(A). The hybrid FETD-SETD solver with *hp*-refinement is employed for this example. The nanoloops are meshed with 2nd order tetrahedrons (*i.e*., *p*-refinement and the FETD solver are employed to capture the curved surfaces of the loops), while the remaining free space in the physical region and the PML region are meshed with 3rd order hexahedron elements (i.e., *h*-refinement and the SETD solver are employed to decrease the DoF).

The two nanoloops are fed with lumped ports located at [−100, −500, −900] nm (active) and [900, 500, 900] nm (passive), respectively. The input voltage source is a 1st order derivative of a BHW pulse with the characteristic frequency of 100 THz. The simulation in the DGTD method uses 5000 time steps with an interval of 0.02 fs, thus the frequency spectrum resolution is 10 THz. The same case is simulated by AEDT and FEKO, in which the frequency sweeps from 10 THz to 250 THz with an interval of 10 THz.

**Figure 4:**
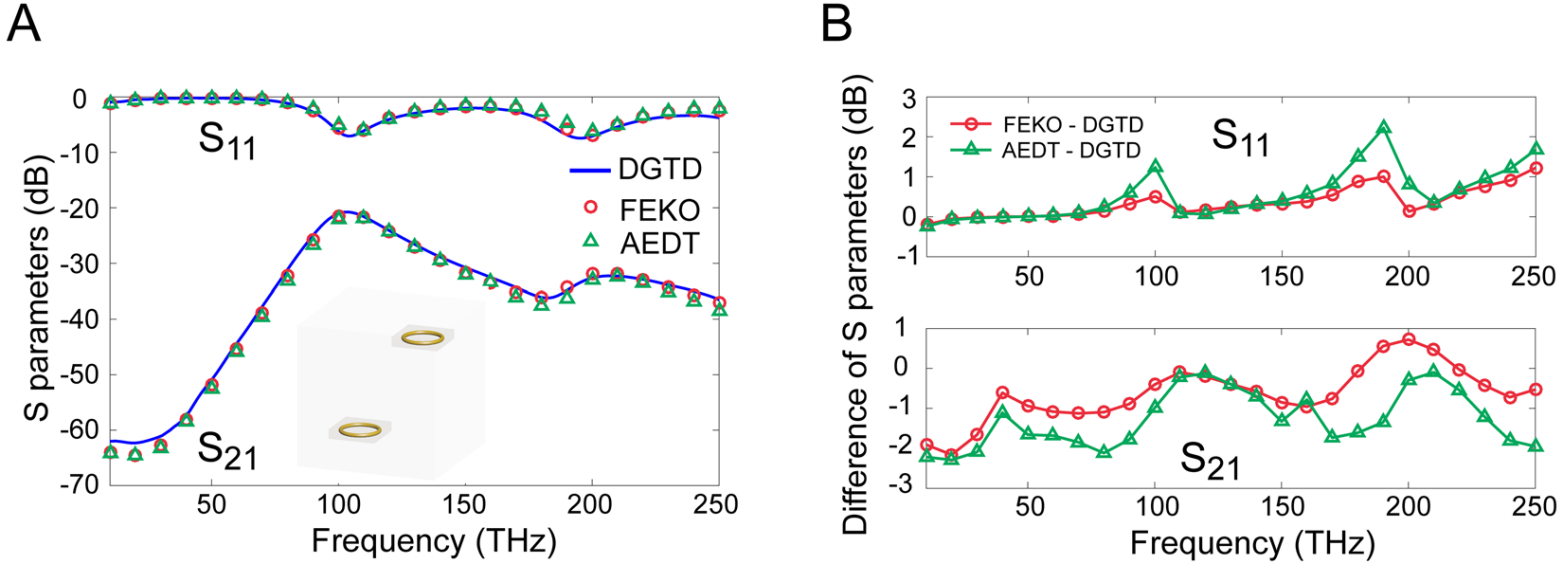
Results of nanoloop coupling example. (**A**) Scattering parameters *S*_11_ and *S*_21_ from the DGTD method, AEDT and FEKO. (**B**) Differences of scattering parameters *S*_11_ and *S*_21_ from the DGTD method, AEDT and FEKO.

The scattering parameters are calculated and compared in Fig. 4(A). The differences between the DGTD method and the commercial software packages are illustrated in Fig. 4(B). Maximal differences for both S_11_ and S_21_ are approximately 2 dB, which indicates that very good agreement is achieved between the three solvers. The computational resource overhead is compared in Table 3. The advantage of the proposed method is obvious since it only requires about one third of the simulation time of its competitors as well as less memory consumption (approximately 60% of FEKO and 28% of AEDT). In addition to the four reasons for this computational gain identified in the above SRR case, an additional advantage of the DGTD method in this coupling case can be attributed to the *hp*-refinement. Coarse high order hexahedron elements can significantly decrease the DoF in the free space far from the nanoloops. Meanwhile the dense tetrahedron mesh for the nanoloops and their surrounding area can capture the curved surfaces. Therefore, this cases can be simulated with high accuracy as well as low computational burden.

**Table 3:** Computational overhead comparison for the nanoloop coupling case between the DGTD method, AEDT and FEKO.

	FEKO	AEDT	DGTD	Gain wrt FEKO/AEDT
Total Time (h)	5.44	5.39	1.59	3.42/3.38
Peak Memory (GB)	2.91	9.76	1.74	1.67/3.52
Time Steps	N/A	N/A	5000	N/A
Time Interval	N/A	N/A	0.02 fs	N/A
Cell Size (nm)	Triangle 20	Tetrahedron 40	Brick 500 Tetrahedron 40	N/A
DoFs (k)	19.52	875.5	445.4	0.044/1.952
Element Number	6656	45914	11561	0.576/3.971

## Conclusions

This paper presents a new DGTD method for simulating the wideband response of nano-architectures comprised of DCP dispersive media. It is based on the framework of domain decomposition. The advanced technologies, such as *hp*-refinement and non-conformal mesh are incorporated to decrease the DoF as well as capture the fine geometries. An E-J collocation scheme is applied to solve the auxiliary differential equations which converts the inversion of the mass matrices to simple vector manipulations. A hybridization of the second order Runge-Kutta and Newmark methods is employed to update the coupled Maxwell’s equations and auxiliary equations in an efficient manner. Various examples are simulated by the newly proposed method and compared to commercial software packages. The results validate the high accuracy of this method and its advantages in computational time and memory consumption. In addition, this approach can be used as a reference in the future study of FETD/SETD/DGTD algorithms based on other complex dispersive models. In upcoming research, the proposed method could, for example, be extended to include periodic boundary conditions for the simulation of photonic metamaterials comprised of DCP media based unit cells.
